# SmartPlus: a computer-based image analysis method to predict continuous-valued vascular abnormality index in Retinopathy of Prematurity

**DOI:** 10.1186/s40942-025-00668-3

**Published:** 2025-04-11

**Authors:** Sayed Mehran Sharafi, Nazanin Ebrahimiadib, Ramak Roohipourmoallai, Afsar Dastjani Farahani, Marjan Imani Fooladi, Golnaz Gharehbaghi, Elias Khalili Pour

**Affiliations:** 1 Translational Ophthalmology Research Center, Farabi Eye Hospital, Tehran University of Medical Sciences, Tehran, Iran; 2 Ophthalmology Department, College of Medicine, University of Florida, Gainesville, FL USA; 3 Department of Ophthalmology, Morsani College of Medicine, University of South Florida, Tempa, FL USA; 4 Retinopathy of Prematurity Department, Farabi Eye Hospital, Tehran University of Medical Sciences, South Kargar Street, Qazvin Square, Tehran, Iran; 5 Clinical Pediatric Ophthalmology Department, UPMC, Children’s Hospital of Pittsburgh, Pittsburgh, USA; 6 Department of Pediatrics, Ali Asghar Children’s Hospital, Iran University of Medical Sciences, Tehran, Iran

## Abstract

Plus disease is characterized by abnormal changes in retinal vasculature of premature infants. Presence of Plus disease is an important criterion for identifying treatment-requiring cases in Retinopathy of Prematurity (ROP). However, diagnosis of Plus disease has been shown to be subjective and there is wide variability in the classification of Plus disease by ROP experts, which is mainly because experts have different cut-points for distinguishing the levels of vascular abnormality. This suggests that a continuous Plus disease severity score may reflect more accurately the behavior of expert clinicians and may better standardize the classification. The effect of using quantitative methods and computer-based image analysis to improve the objectivity of Plus disease diagnosis have been well established. Nevertheless, the current methods are based on categorical classifications of the disease severity and lack the compatibility with the continuous nature of the abnormal changes in retinal vasculatures. In this study, we developed a computer-based method that performs a quantitative analysis of vascular characteristics associated with Plus disease and utilizes them to build a regression model that outputs a continuous spectrum of Plus severity. We evaluated the proposed method against the consensus diagnosis made by four ROP experts on 76 posterior ROP images. The findings of our study indicate that our approach demonstrated a relatively acceptable level of accuracy in evaluating the severity of Plus disease, which is comparable to the diagnostic abilities of experts.

## Introduction

ROP is a leading cause of childhood blindness worldwide, characterized by abnormal retinal vascular development at the boundary of vascularized and avascular peripheral retina [1–3]. The incidence of ROP is approximately 9% in developed countries and 12% in developing countries, with the risk of blindness reaching 44% in low- and middle-income countries due to limited access to neonatal intensive care and retinal screening [4, 5]. Reports indicate that the global prevalence of childhood and adulthood vision loss due to ROP continues to increase [6].

“Plus disease” defined by abnormal vascular dilation and tortuosity, is a critical indicator of severe ROP and a key factor in determining the need for treatment. While timely interventions such as laser photocoagulation and intravitreal bevacizumab can effectively treat ROP [7–9], accurate diagnosis remains challenging due to high inter-examiner variability among even experienced clinicians [10–13]. This variability stems from the lack of standardized thresholds for assessing vascular abnormalities, with experts often using different cut-points to diagnose Plus disease [14, 15].

To address these challenges, there is growing interest in artificial intelligence (AI) and computer-based methods for automated ROP diagnosis. However, most existing methods rely on discrete classifications (e.g., Normal, pre-Plus, or Plus), which fail to capture the continuous spectrum of vascular changes in ROP [14]. In this study, we propose a computer-based method that predicts a continuous severity score for Plus disease, offering a more objective and nuanced approach to ROP diagnosis. Our method leverages a modified U-Net architecture for vessel segmentation and incorporates vessel density as a novel feature, alongside traditional markers like dilation and tortuosity. This approach not only improves diagnostic accuracy but also aligns with the real-world practices of ROP experts, who assess severity on a spectrum rather than rigid categories.

Additionally, our study is conducted on a domestic dataset from Farabi Eye Hospital, a leading center for ROP in Iran, providing region-specific insights into ROP diagnosis and treatment. By addressing the limitations of existing methods and leveraging local data, our work contributes to the global understanding of ROP while offering practical solutions for resource-limited settings. The integration of this method into telemedicine platforms could expand access to ROP screening and reduce the burden on healthcare systems, ultimately improving outcomes for premature infants at risk of blindness.

## Methods

### Ethics

The study adhered to the principles outlined in the Declaration of Helsinki and received approval from the institutional review board of Tehran University of Medical Sciences. Written informed consent was obtained from the parents of all patients involved in the study, granting permission for imaging and participation in the research.

### Subjects and reference standard diagnosis

A database of 76 wide-angle posterior retinal images was established, each corresponding to different preterm infants acquired during routine clinical care. The subjects included 27 female and 49 male infants with an average birth weight of 1305 g ± 427 g and an average gestational age of 29.3 weeks ± 3 weeks. These infants were examined at Farabi Eye Hospital in Tehran, Iran, between January 1, 2019, and December 30, 2020, and met published criteria for ROP screening. All images were captured using a RetCam device with dimensions of 1200 × 1600 pixels. Four ROP expert graders independently categorized selected images into five levels of severity: “Normal,” “pre-Plus,” “Plus1,” “Plus2,” and “Plus3,” assigning integer scores of 1 through 5, respectively. A reference standard diagnosis (RSD) was defined for each image as the majority score given by the four experts. In cases of discordant scores, these were adjudicated in group sessions involving all four experts to determine the standard diagnosis. A web-based user interface was created to streamline the grading process and eliminate potential biases.

### Image selection and analysis

Difficulties associated with retinal imaging in infants, such as smaller eyes, less developed pupils, pressure on the globe during image capture, poor fixation, motion blurring, and uneven illumination, often result in low-quality images [16, 17]. To address concerns regarding image quality, an expert excluded low-quality images from the dataset.

### Vessel segmentation

**Fig. 1:**
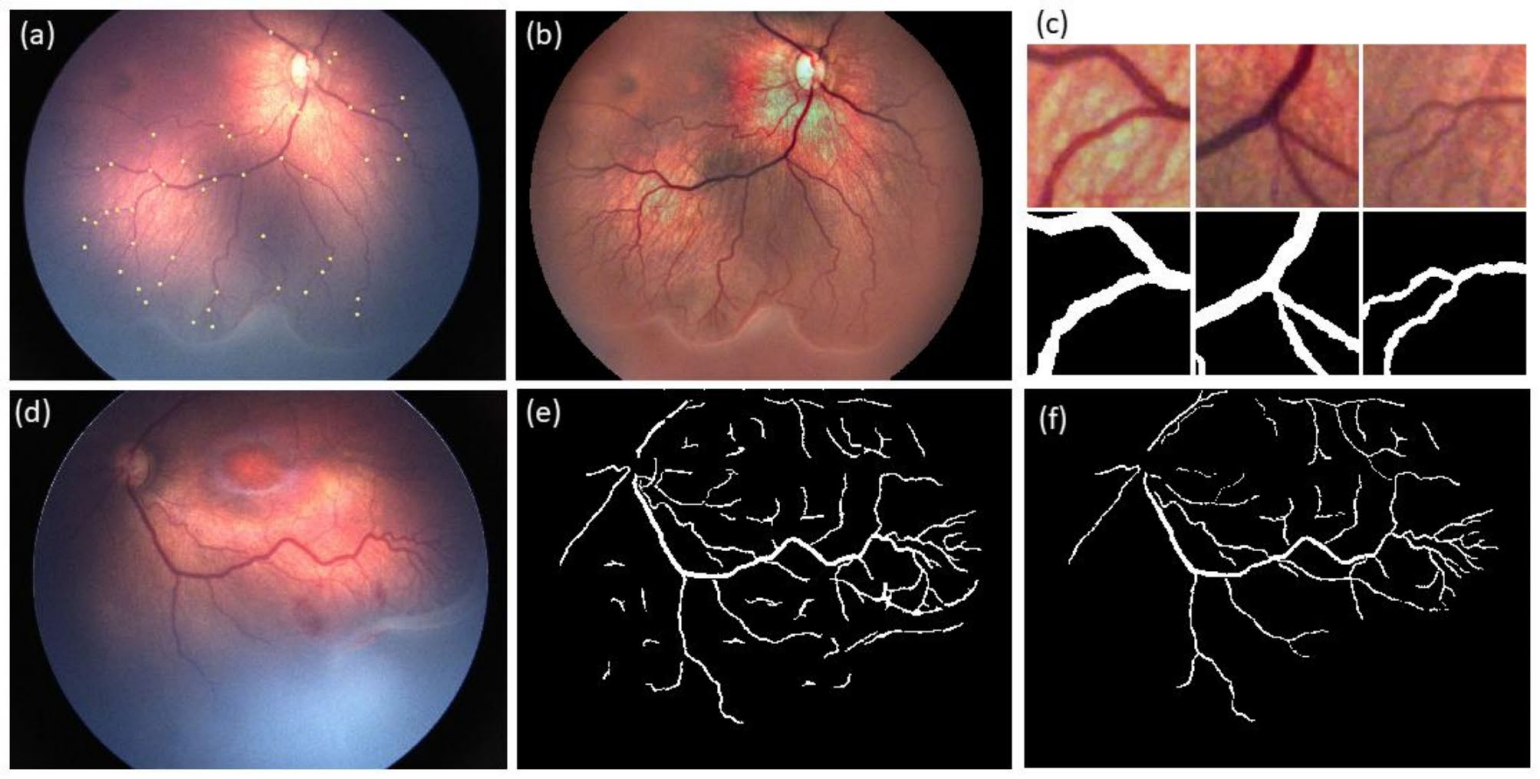
Outputs of our vessel segmentation method. (**a**) Selected bifurcation points marked by yellow dots used as the centers of the patches in a sample image. (**b**) Preprocessed version of the image, ”a” using CLAHE algorithm. (**c**) Three patches and their corresponding masks extracted from image “b”. (**d**) A sample image input to the trained network. (**e**) Predicted vessel mask of image “d” by the network. (**f**) Vessel mask after making correction on the output of the network by an expert

Vessel segmentation was performed using a modified U-Net encoder/decoder network [18]. For training purposes, 80 images were enhanced using the Contrast-Limited Adaptive Histogram Equalization (CLAHE) and were corrected for uneven illumination. Vessel masks were created by experts using a Graphical User Interface (GUI) previously developed for semi-automated retinal vessel segmentation in fundus images [19]. The network was trained using 2,736 image patches of size 128 × 128 pixels along with their corresponding masks. Patches were made around the centers of randomly selected bifurcation points. This method helped creating an efficient data set of image patches and avoid picking less informative patches. 80% of the patches were used for training, and the remaining 20% were used for validation. Unlike conventional U-Net architectures that use pixel-wise cross-entropy loss functions, we employed the Tversky loss function [20], which better measures overlap between segmented regions. Morphological opening with a structuring element of 200 pixels was applied to eliminate small false-positive regions on the mask produced by the network. This approach achieved a segmentation accuracy of 91% in terms of BF score on a separate test dataset of 20 images. The GUI was used to correct errors in the masks produced by the automatic segmentation and to mark optic disc borders (Figure 1).

### Quantification of vessel characteristics

Arterial tortuosity is a key component of the ROP international classification system for diagnosing Plus disease [21]. We quantified vessel tortuosity using the squared-derivative-curvature method [22], which estimates tortuosity as the integral of the square of the derivative of curvature divided by the arc length of the vessel segment. This method is more accurate than the arc-to-chord ratio method, as it accounts for the entirety of the vessel network geometry [23]. Using the mentioned method in this study, we were also able to calculate point-based curvature through the vessel segments. It is shown in recent studies that point-based curvature of the vessels is also significantly increased in Plus disease [24, 25]. Detailed explanation on the calculation of the curvature-based tortuosity can be found in Sharafi et al. [19, 24]. Additionally, vessel dilation was quantified by calculating the average vessel diameter within a region extending three-disc diameters (3DD) away from the optic disc border.

In previous studies, the density of retinal vessels has been extensively addressed as a potential marker of ROP [26–28], especially in relation to Plus disease [24, 28]. Thus, in addition to assessing tortuosity and dilatation, we also looked at the vessels’ density as a potential indicator of Plus disease. The ratio of the number of pixels located on the vessels over the remaining pixels in the entire vascularized region was used to calculate the vessels density for each image.

Statistical analyses were performed to assess the effectiveness of each feature in distinguishing severity levels.

### Regression model

**Fig. 2:**
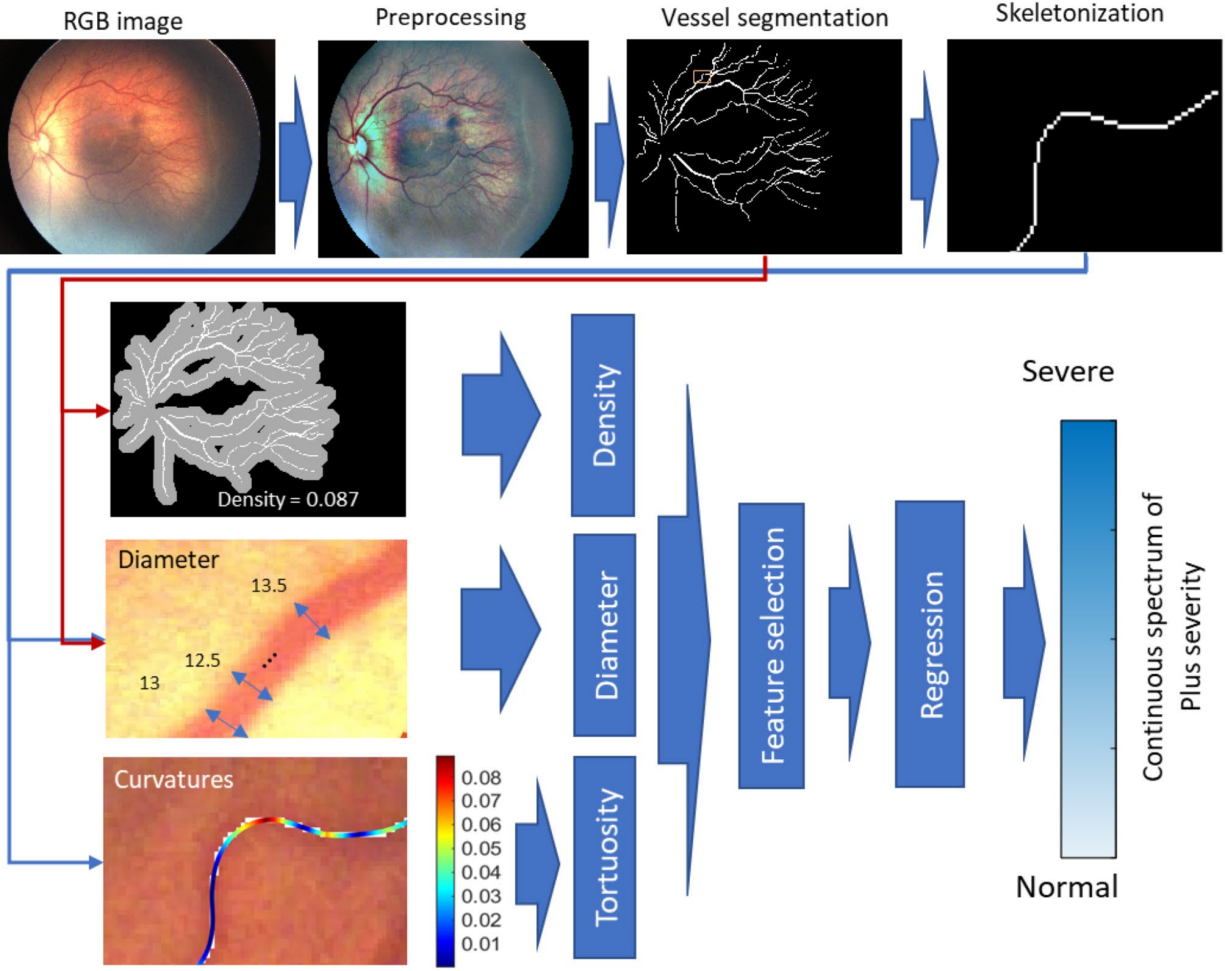
Schematic of the proposed method and sample outputs at each step. Skeletonization, and values for diameter and curvature are shown for a small vessel segment within the image. Curvature values at each point of the segment are shown by a colormap. (Modified from Sharafi et al. [24])

The main objective of this study was to develop an algorithm to predict a continuous vascular abnormality index as a measure of Plus severity. Initially, a set of 10 features characterizing aspects of vessel tortuosity, dilation, and density across various retinal regions was retrieved. To prioritize features with greater discriminatory power, Neighborhood Component Analysis (NCA) for regression was utilized [29]. Ultimately, a linear regression model was trained to predict the severity score of Plus disease using four selected features: F1) maximum vessel diameter within 3DD, F2) average tortuosity of vessel segments in 3DD, F3) vessel density across the entire image, and F4) average of the top 1% point-based curvature values in the entire image. Figure 2, depicts a schematic representation of the suggested technique, together with sample outputs observed at each step.

## Results

### Inter-expert variability and experts’ agreement

**Fig. 3:**
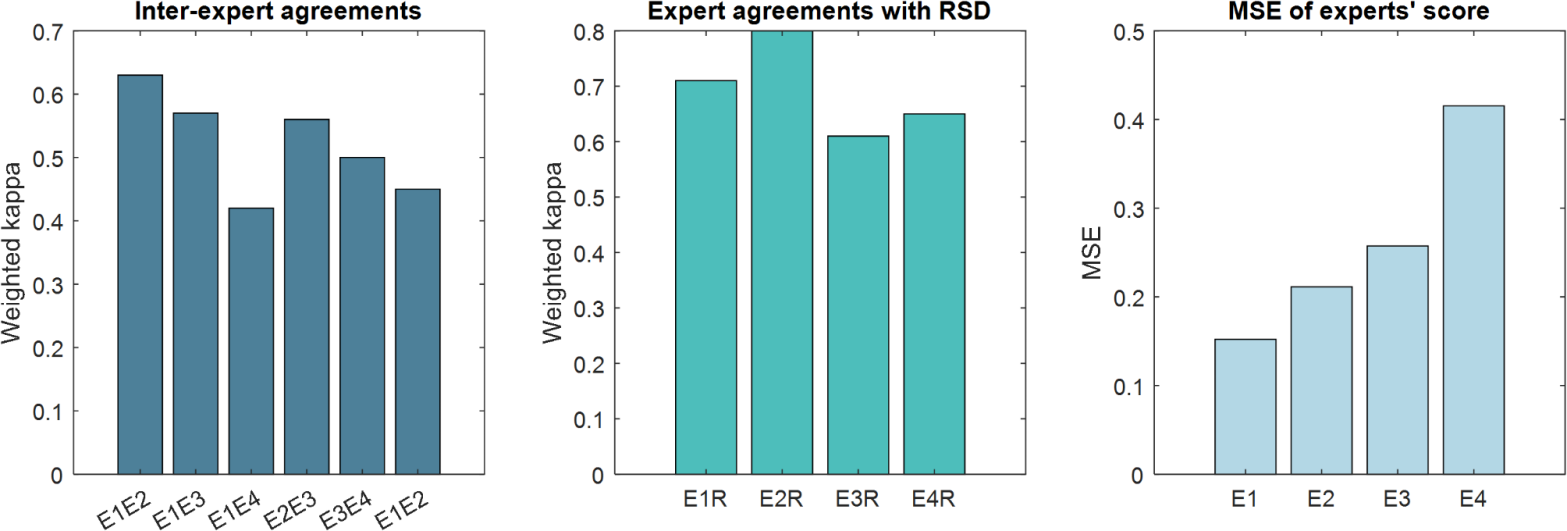
Inter-expert agreement, individual expert agreement with RSD, and MSE against the average score. The figure shows the weighted kappa (linear) for inter-expert agreement (left chart) and individual expert agreement with RSD (middle chart), along with the MSE values (right chart) for individual experts. The x-axis represents the grader/expert pairs (E1E2, E1E3, etc.) and individual experts (E1, E2, etc.), while the y-axis represents the weighted kappa values and MSE scores

To evaluate the agreement among the four ROP experts in grading the images, we calculated weighted kappa (linear) statistics for both inter-expert agreement and individual expert agreement with the reference standard diagnosis (RSD). The inter-expert agreement, as measured by weighted kappa, ranged from 0.42 to 0.63, indicating moderate to substantial agreement among the graders. The individual expert agreement with RSD showed higher weighted kappa values, ranging from 0.61 to 0.8, suggesting strong agreement between individual experts and the reference standard. The MSE values for individual experts ranged from 0.1521 to 0.4153, with G1 showing the lowest MSE, indicating the highest consistency with the average score. To visualize these results, Figure 3 illustrates the inter-expert agreement, individual expert agreement with RSD, and the MSE values. The figure highlights the variability in expert diagnoses and the influence of the different cut-points for assessing Plus severity in a categorical scoring systems.

To each level of the severity, an integer number from 1 to 5 was assigned as follow: “Normal” = 1, ‘’pre-Plus” = 2, ‘’Plus1” = 3, ‘’Plus2” = 4, Plus3” = 5. Then an average score for each image was calculated based on the assigned numbers.

**Fig. 4:**
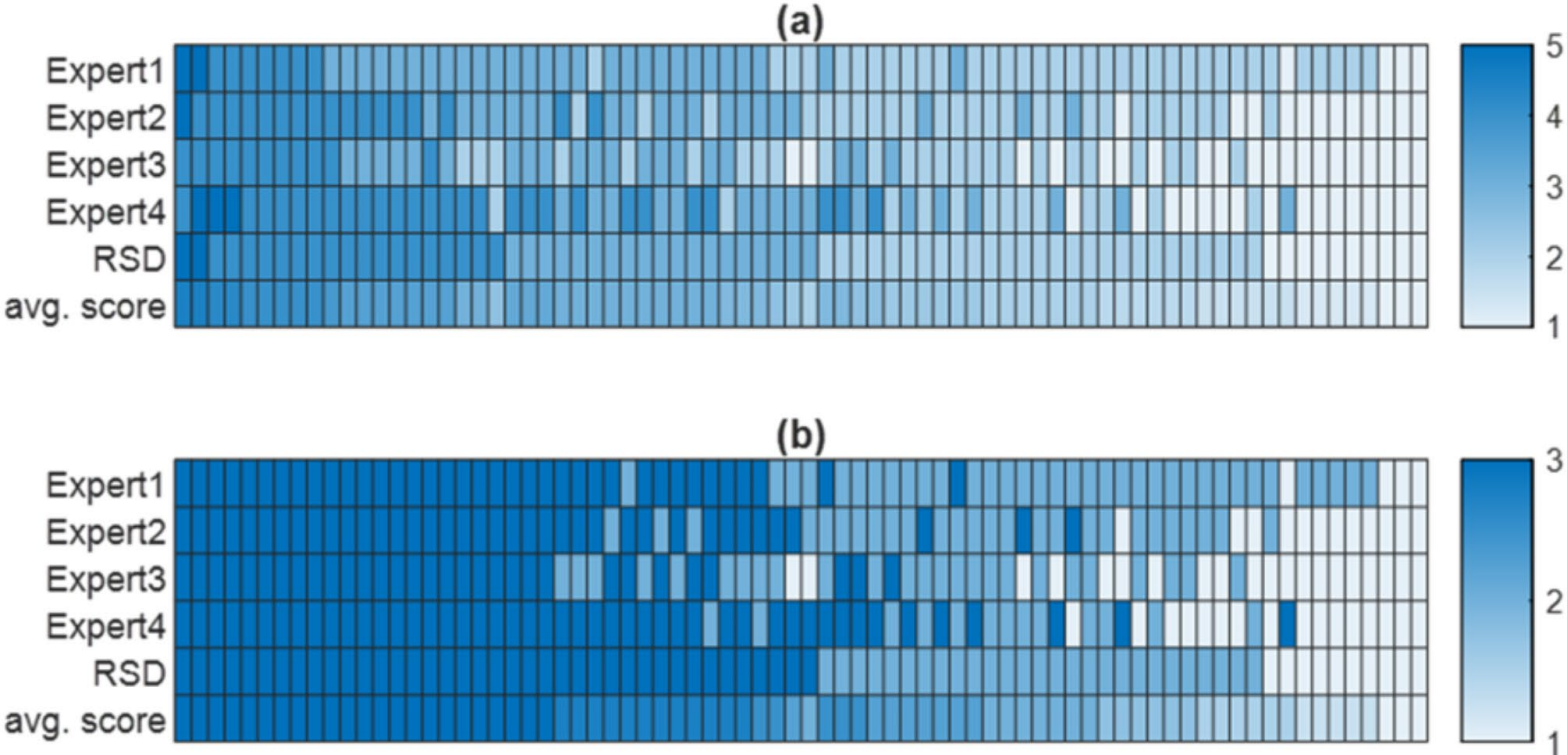
Diagnostic classification of Plus disease by 4 experts based on 5-level (**a**) and 3-level (**b**) classification methods. (**a**) Each column represents an image ranked as 1(Normal), 2(pre-Plus), 3 (Plus1), 4 (Plus2), 5 (Plus3). (**b**) Each column represents an image ranked as 1 (Normal), 2 (pre-Plus), 3 (Plus). RSD depicts the Reference Standard Diagnosis for each image, and avg. score depicts continuous-valued scores based on average of the expert classifications

To make an overall comparison between our 5-level grading method and the conventional 3-level grading method, we extracted a 3-level grading data from our original 5-level grading data by considering grades given as either of “Plus1”, “Plus2”, or “Plus3” to an aggregated “Plus” grade. Figure 4a and b demonstrate the range of diagnoses for individual images, ordered by RSD for 5-level and 3-level grading methods respectively. Each column is associated with an individual image ranked as most severe (dark blue) to less severe (light blue) by each of the 4 experts.

### Impact of vascular measures

**Fig. 5:**
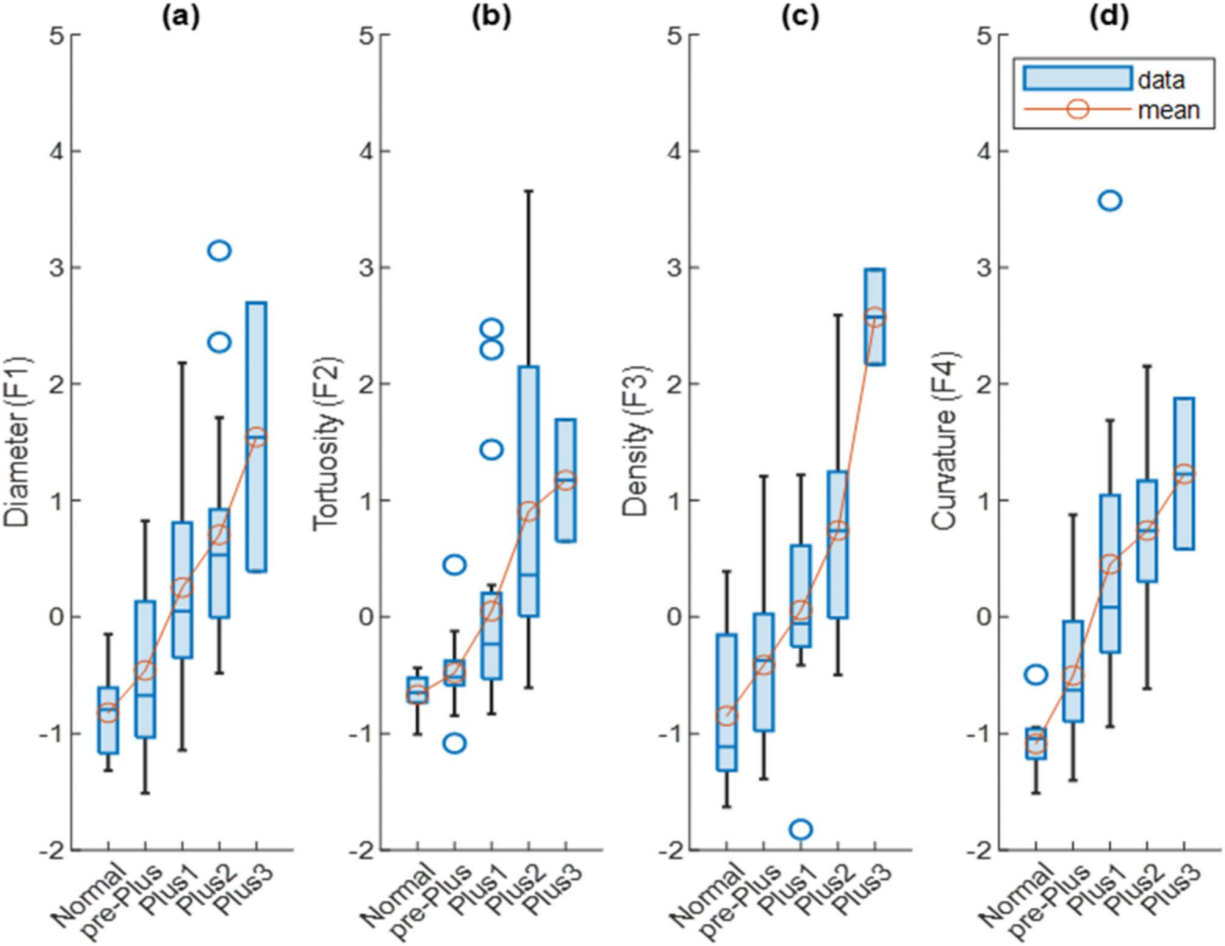
Comparison between the values of each extracted feature, (i.e. F1 to F4) and the five levels of the reference standard (values are standardized). **a**) Maximum of the vessel diameter in a region including 3 diameters away from the optic disk border (3DD). **b**) Average of the tortuosity of the vessel segments in 3DD region. (**c**) Vessel density measured at the vascular region of the retina. (**d**) Average of top 1% point-based curvature values. Blue circles illustrate the outlier datapoints. Red circles depict mean value of the features in each level of disease severity

In accordance with the preceding section, four image features F1 through F4 were used to train a regression model to predict a continuous-valued severity score. Results of comparing the different groups of images based on the mentioned features are shown in Figure 5. To show the efficiency of each feature in discerning any pairs of severity levels, t-tests between mutual levels of severity were conducted. As a complementary data to Figure 5., Table 1 summarizes the p-values associated with each of the features in discerning each pairs of severity levels. Rows of the table includes pairs of the severity levels and each column corresponds to a feature.

**Table 1:** Results of t-tests applied on mutual levels of plus severity based on the four selected features. P-values corresponding to significant differences are depicted as follow: *p* < 0.05: *; *p* < 0.01: **; *p* < 0.001: ***

		*p*-values			
Severity levels		Diameter	Tortuosity	Density	Curvature
‘Normal’	‘pre-Plus’	0.1449	0.0518	0.1059	0.0031 **
‘Normal’	‘Plus1’	0.0010 **	0.0293*	0.0017**	0.0001 ***
‘Normal’	‘Plus2’	6.0e-05 ***	0.0006 ***	5.0e-05 ***	7.9e-08***
‘Normal’	‘Plus3’	0.0005 ***	1.0e-05 ***	7.0e-05 ***	2.0e-05 ***
‘pre-Plus’	‘Plus1’	0.0046 **	0.0099 **	0.0290*	0.0002 ***
‘pre-Plus’	‘Plus2’	4.0e-05 ***	1.6e-06***	2.0e-05 ***	9.0e-08 ***
‘pre-Plus’	‘Plus3’	0.0017 **	6.06e-08 ***	4.7-e06 ***	0.0003 ***
‘Plus1’	‘Plus2’	0.1438	0.0262 *	0.0126 *	0.3502
‘Plus1’	‘Plus3’	0.0783	0.1331	6.0e-05 ***	0.3371
‘Plus2’	‘Plus3’	0.2825	0.7749	0.0130 *	0.4013

### Regression analysis

**Fig. 6:**
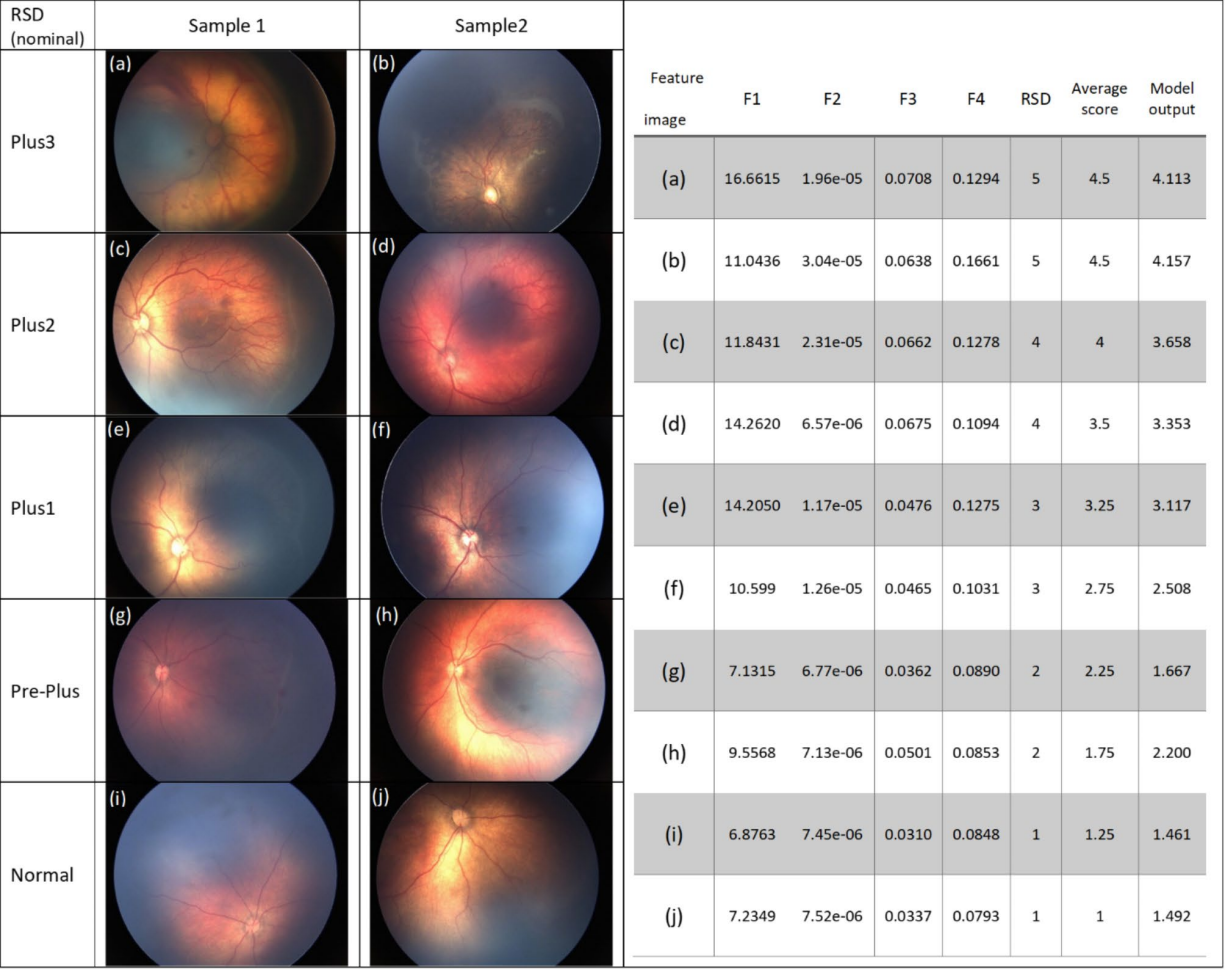
Feature values, RSD, average scores, and the model outputs corresponding to 10 sample images in the dataset (two samples per each severity level are shown)

Using selected features, F1 to F4, we trained a linear regression model to predict *Plus severity index* as a range of continuous values from 1 (less severe) to 5 (most severe). Figure 6 shows 10 sample images, their corresponding feature values, RSDs, the average scores, and the regression model’s out puts (two samples per each severity level are shown).

**Fig. 7:**
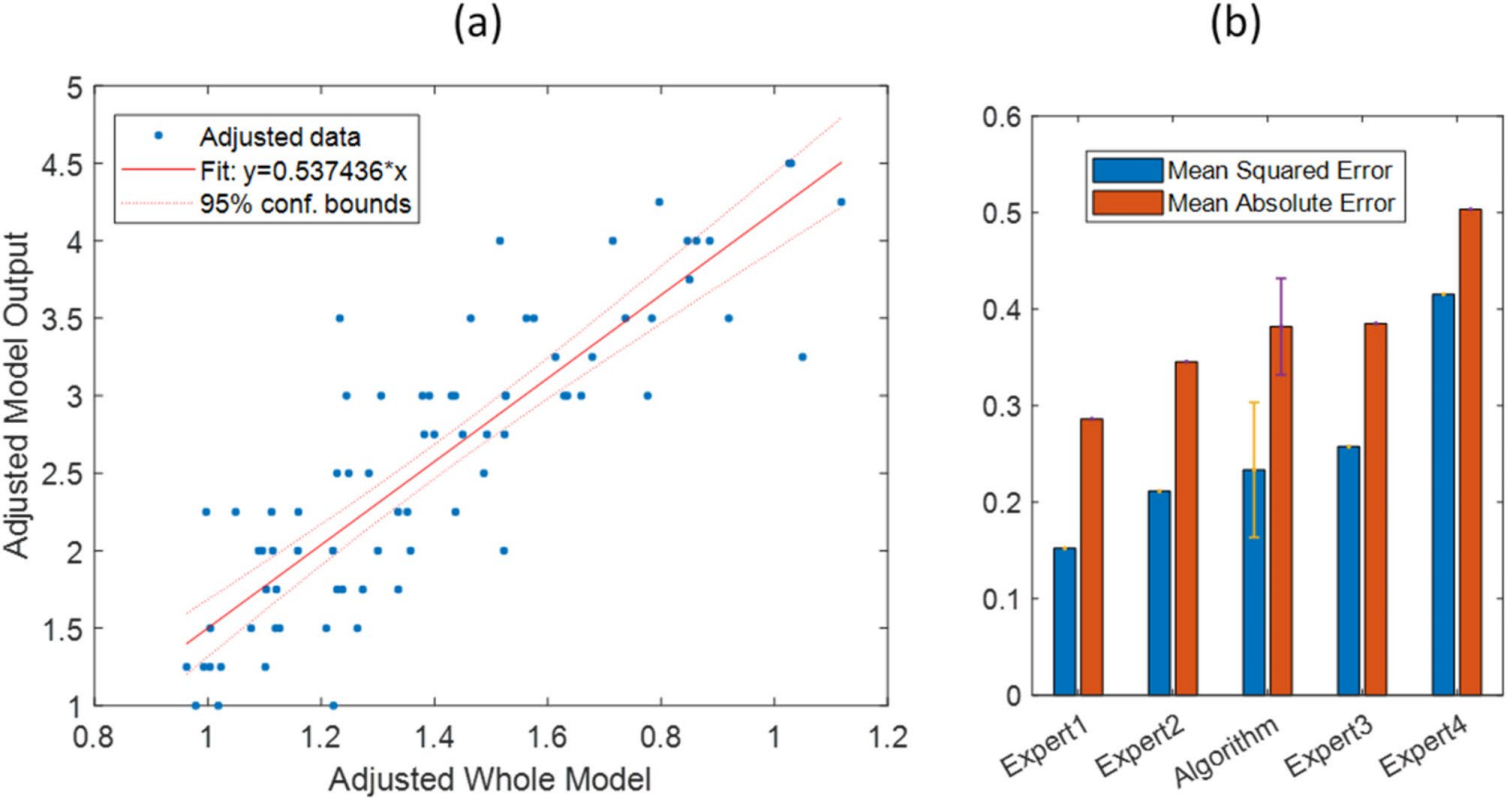
(**a**) Added Variable Plot for the proposed regression model. Fit-line equation, adjusted-value data, and confidence bound are shown in the legend. (**b**) A comparison between the losses of the algorithm and experts’ diagnoses against the images’ average scores as a gold standard. Error bars shows the standard deviation of the MSE and MAE values yielded for the regression model

To assess the effectiveness of the suggested regression model, we employed an Added Variable Plot to illustrate the association between the model’s output and the predictor variables. The resulting plot is depicted in Figure 7-a. Slope of the fit line and the confidence interval bound shows the explanatory power of the regression model.

Moreover, we evaluated the accuracy of our regression model using a 5-fold cross validation and calculating MSE and Mean Absolute Error (MAE) of the model output against the average scores as a gold standard. MSEs and MAEs of each expert’s diagnosis were also calculated and compared with the MSE and MAE of the regression model respectively.

The comparison is briefly presented in Figure 7-b. According to the data presented, MSE and MAE of the regression model are 0.23 ± 0.07 and 0.38 ± 0.05 respectively. This indicates that the model outperformed two of the experts in terms of its predictive performance.

## Discussion

The diagnosis of ROP remains challenging due to its subjective nature, particularly in the assessment of Plus disease. While wide-field retinal imaging, such as RetCam, has improved access to ROP screening, diagnostic variability persists even among experts [12, 13]. This variability arises from differences in the interpretation of vascular abnormalities, such as tortuosity and dilation, and the lack of standardized thresholds for diagnosing Plus disease. Our study addresses these challenges by proposing a computer-based method that quantifies vascular abnormalities and predicts a continuous severity score, offering a more objective approach to ROP diagnosis.

While quantification of vessel characteristic is well-established in previous studies, our study introduces several technical innovations tailored for the specific application of Plus disease severity assessment in ROP. First, we modified the U-Net architecture by employing the Tversky loss function instead of the traditional pixel-wise cross-entropy loss. This modification improved the segmentation accuracy when applied to our dataset. Additionally, we implemented an improved preprocessing step that not only enhanced the contrast of vessels against background retina, but also corrects uneven illumination, ensuring more reliable vessel segmentation even in low-contrast regions.

Another key contribution of our method is the incorporation of vessel density as a feature in the regression model. While vessel dilation and tortuosity are the established criteria for diagnosing Plus disease, our findings reveals that vessel density (specially vessel structure located closed to the vascularized side of ridge lines) can unintentionally influence expert judgments. This observation highlights the potential for additional vascular features to improve the accuracy of automated systems. It further validates the results of earlier studies concerning the alterations in vessel density in ROP [26–28]. Although vessel density is not currently a standard criterion, its inclusion in our model demonstrates how supervised AI systems can capture subtle patterns in expert-labeled data, even beyond the established diagnostic criteria. We are currently conducting further studies to explore how experts may be influenced by vessel anomalies beyond dilation and tortuosity, which could lead to new insights into diagnostic biases and improve the robustness of automated systems.

In this study we introduced a 5-level grading system including “Normal”, “pre-Plus”, “Plus1”, “Plus2”, and “Plus3” and assigned numbers 1 to 5 to each of the levels to calculate an average score of severity for each image. The rationale for implementing a 5-level severity grading system instead of 3-level system, can be comprehended by considering the following factors:

1-The existence of Plus disease in ROP signifies a continuous range of vascular abnormalities. This implies that there is considerable variation in the severity of the condition among patients, and a three-level measurement may not sufficiently encompass this range of variability [30].

2. The implementation of a multi-level grading system has the potential to enhance the precision and dependability of diagnostic procedures. It enables a more nuanced measure of disease progression, which can be essential in deciding the best treatment strategy [12, 16, 30].

Moreover, this method of grading helped us to calculate a more accurate average score of severity as a gold standard for training a regression model compared to a 3-level grading system in which Normal, pre-Plus, and Plus levels are assigned numbers 1,2, and 3.

A significant contribution of our study is the development of a continuous severity index for Plus disease through a regression model. In contrast to the majority of current methods that depend on discrete classifications (e.g., Plus, pre-Plus, or Normal), our approach reflects the continuous nature of vascular changes in ROP. This is particularly important because Plus disease progression and treatment response are gradual phenomena, and a continuous index provides a more nuanced and accurate representation of disease severity [30]. By predicting a continuous score, our model aligns more closely with the real-world diagnostic practices of ROP experts, who often assess severity on a spectrum rather than rigid categories. This innovation offers a more flexible tool for monitoring disease progression and treatment outcomes.

Another contribution of our study is the use of a domestic dataset acquired from Farabi Eye Hospital, a leading center for ROP in Iran. This dataset allowed us to evaluate the agreements among experienced ROP specialists in a specific geographical region, where ROP prevalence, screening practices, and imaging setups may differ from those in other parts of the world. Local studies like ours are crucial for understanding regional variations in ROP diagnosis and treatment, as they reveal unique challenges and opportunities for improving clinical workflows. By leveraging this dataset, we were able to develop a model that is tailored to the specific needs of our clinical setting, while also contributing to the global understanding of ROP. Our findings underscore the importance of region-specific research in advancing ROP screening and diagnosis, particularly in resource-limited settings where access to experienced specialists is limited.

Our findings are consistent with previous studies that have explored automated methods for ROP diagnosis. Table 2 presents a comparison of our approach with the well-known method introduced for Plus disease detection in recent years. Our approach demonstrates uniqueness compared to other methods as it has established a five-level grading system that produces a continuous-valued Plus severity index via a regression model.

**Table 2:** A comparison between our method and similar methods established for plus disease detection

Criteria Method	Vessel segmentation	Feature extraction	Vessel feature (s)	Tortuosity measurement	Model Out put	Grading system	Supervise /unsupervised	Discrete/continuous	Prediction model
ROPTool [31, 32]	“ridge/ valley traversal” method	HCF	T, D	ACR	T-index, D-index	N/A	N/A	N/A	N/A
Tian, P. (2016) [33]	manual	HCF	Multiple features related to T	SDC	Severity index	3-level	Un-supervised	Continuous	Clustering by similarity
i-ROP [25]	manual	HCF	Multiple features related to T and D	SDC	Plus/pre-Plus/normal	3-level/ 9-level	Supervised	Discrete/ Continuous	SVM classification
i-ROP-DL [14]	CNN (U-Net)	DL	N/A	N/A	Plus/pre-Plus/normal	3-level/ 9-level	Supervised	Discrete/ Continuous	CNN
Our method	CNN (a modified U-net)	HCF	T, D, C, Density	SDC	Vessel abnormality index	3-level/ 5-level	Supervised	Continuous	Linear regression

T = Tortuosity; D = Dilation; C = Curvature; SDC = squared-derivative-curvature, ACR = Arc-to-cord ratio; CNN = convolutional neural network; SVM = support vector machine

Despite the mentioned contributions, our study has some limitations. First, the dataset was relatively small and derived from a single clinical site, which may limit the generalizability of the results. Future studies should incorporate larger, more diverse datasets to improve the model’s robustness and applicability across different populations. Second, the algorithm’s performance was evaluated using high-quality images, and its effectiveness on lower-quality images remains uncertain. This is a critical consideration for real-world applications, where image quality can vary significantly. Finally, factors such as imaging angle, pressure from the RetCam contact camera, and the administration of mydriatics may introduce measurement bias, which should be addressed in future work.

In conclusion, in the current study we developed a novel computer-based method for diagnosing Plus disease in ROP by outputting a continuous severity score, addressing the limitations of traditional discrete classifications. Key contributions include a modified U-Net architecture with Tversky loss and CLAHE preprocessing, achieving 91% segmentation accuracy, and the introduction of vessel density as a novel feature that improves regression accuracy. Unlike existing methods, our model provides a continuous severity index (1 to 5), aligning with the gradual progression of vascular abnormalities and enabling precise monitoring. Using a domestic dataset from Farabi Eye Hospital, we provided regional insights into ROP diagnosis, contributing to global understanding. The regression model outperformed two of four experts, with selected features demonstrating strong discriminatory power, particularly between pre-Plus and Plus1 levels. Future work should incorporate larger datasets and assess diagnostic biases. This approach enhances diagnostic accuracy, reduces expert variability, and supports clinical decision-making. Integrated into telemedicine platforms, it could expand access to ROP screening, improving outcomes for at-risk infants.

## Data Availability

No datasets were generated or analysed during the current study.
